# Diagnostics of Internal Medicine

**Published:** 1910-01

**Authors:** 


**Diagnostics of Internal Medicine.** By **Glentworth R. Butler,** M.D., Physician-in-Chief to the Methodist Episcopal Hospital, Brooklyn. Third edition. Octavo, pp. 1227. Illustrated. New York and London: D. Appleton & Co. 1909. (Cloth, $6.00.)

This treatise is one of the best of its class. In this edition the author has brought the work well abreast of the latest thought and best literature on the subject. No important diagnostic improvement seems to have been overlooked.

The arrangement of the book into two parts makes it very convenient for reference. The spelling of the words "color" and "odor," and some others throughout the book is not according to American usage, as the addition of the letter "u" to the words gives them an unfamiliar appearance. The book taken as a whole, however, is a credit to the author who is one of our ablest diagnosticians. J. A. R.

